# StackGlyEmbed: prediction of N-linked glycosylation sites using protein language models

**DOI:** 10.1093/bioadv/vbaf146

**Published:** 2025-06-28

**Authors:** Md Muhaiminul Islam Nafi, M Saifur Rahman

**Affiliations:** Department of Computer Science and Engineering, Bangladesh University of Engineering and Technology, Dhaka 1000, Bangladesh; Department of Computer Science and Engineering, United International University, Dhaka 1212, Bangladesh; Department of Computer Science and Engineering, Bangladesh University of Engineering and Technology, Dhaka 1000, Bangladesh

## Abstract

**Motivation:**

N-linked glycosylation is one of the most basic post-translational modifications (PTMs) where oligosaccharides covalently bond with Asparagine (N). These are found in the conserved regions like N-X-S or N-X-T where X can be any residue except Proline (P). Prediction of N-linked glycosylation sites has great importance as these PTMs play a vital role in many biological processes and functionalities. Experimental methods, such as mass spectrometry, for detecting N-linked glycosylation sites are very expensive. Therefore, the prediction of N-linked glycosylation sites has become an important research field.

**Results:**

In this work, we propose StackGlyEmbed, a stacking ensemble machine learning model, to computationally predict N-linked glycosylation sites. We have explored embeddings from several protein language models and built the stacking ensemble using Support Vector Machine (SVM), Extreme Gradient Boosting (XGB) and *K*-nearest Neighbor (KNN) learners in the base layer, with a second SVM model in the meta layer. StackGlyEmbed achieves 98.2% sensitivity, 92.5% balanced accuracy, 89.1% F1-score and 82.6% Matthew’s correlation coefficient in independent testing, outperforming the existing state-of-the-art methods.

**Availability and implementation:**

StackGlyEmbed is freely available at: https://github.com/nafcoder/StackGlyEmbed.

## 1 Introduction

Post-translational modifications (PTMs) refer to side chain modification of amino acids in proteins after their biosynthesis (Ramazi and Zahiri 2021). N-linked glycosylation is one of the most basic PTMs where Asparagine (N) covalently bonds with oligosaccharides, thus producing N-glycans. This PTM appears in highly conserved areas like N-X-S or N-X-T (Gavel and Heijne 1990, Kowarik *et al.* 2006). Here, in N-X-[S/T], the 1st position will be Asparagine (N), the 2nd position can be any residue except Proline (P) and the 3rd position can be Serine (S) or Threonine (T). N-linked glycosylation is necessary for the structure, stability and functionality of glycoproteins in both prokaryotic and eukaryotic organisms. It plays a crucial role in protein folding and stability, biological functions, immune responses, cell-to-cell communication and clinical diagnosis. N-X-[S/T] does not guarantee that the site is N-linked glycosylated (Petrescu *et al.* 2004, Nita-Lazar *et al.* 2005, Zielinska *et al.* 2010, Schulz 2012). Other factors like distance to the next glycosylated site, and neighboring residues of the potential sequon can also have an effect on its being glycosylated or not. For N-linked glycosylation to take place, having N-X-[S/T] sequon is therefore necessary but not sufficient (Gavel and Heijne 1990, Petrescu *et al.* 2004, Nita-Lazar *et al.* 2005, Wacker *et al.* 2006).

Methods like radioactive chemical labeling (Slade *et al.* 2014) and mass spectrometry (Agarwal *et al.* 1969, Medzihradszky 2005) are utilized for the identification of N-linked glycosylation. As these methods are very expensive, computationally predictive methods have been created to predict N-linked glycosylation over the years. Gupta and Brunak (2001) crafted glycosylation site prediction methods for N-linked, O-linked GalNAc (mucin-type) and O-β-linked GlcNAc (intracellular/nuclear) glycosylations using Artificial Neural Networks (ANN) that examined correlations in the local sequence context and surface accessibility. Subsequently, Gupta *et al.* (2004) designed NetNGlyc, an ANN-based method, that predicted N-glycosylation sites in protein sequences. Chauhan *et al.* (2012) developed GlycoPP, which is an SVM-based prediction model trained on O- and/or N-glycosylated proteins from six different archaeal and bacterial phyla. The features were different sequential properties. Chuang *et al.* (2012) proposed NGlycPred that utilized both structural and residue pattern information using the Random Forest algorithm on a set of N-linked glycosylated protein structures. Chauhan *et al.* (2013) created GlycoEP, a support vector machine (SVM) classifier model, which predicted N-linked, C-linked, and O-linked glycosites in eukaryotic glycoproteins using two larger datasets, namely, standard and advanced datasets.

Li *et al.* (2015) constructed GlycoMine using the random forest (RF) algorithm for computer-based identification of C-linked, N-linked, and O-linked glycosylation sites belonging to the human proteome. Pitti *et al.* (2019) built N-GlyDE, a two-stage prediction tool that predicted N-linked glycosylation sites in human proteins. The first stage used a protein similarity voting algorithm, and the second stage used SVM. Taherzadeh *et al.* (2019) proposed SPRINT-Gly that used deep neural network for predicting N-linked glycosylation sites for humans and mice. Pugalenthi *et al.* (2020) developed Nglyc, a Random Forest Method, for the prediction of N-Glycosylation sites in proteins related to humans and mice. Chien *et al.* (2020) manufactured N-GlycoGo, a predicting tool, using XGBoost for predicting N-Glycosylation sites on humans and mouse glycoproteins. Pakhrin *et al.* (2021) designed DeepNGlyPred, a deep learning-based approach, that trained and tested on the human proteome datasets (N-GlyDE and N-GlycositeAtlas). Pakhrin *et al.* (2023) developed LMNglyPred, a deep learning-based approach, to predict N-linked glycosylated sites in human proteins (N-GlyDE and N-GlycositeAtlas). They used embeddings from a pre-trained protein language model [ProtT5-XL-U50 (Elnaggar *et al.* 2022)] as features. Hou *et al.* (2023) designed EMNGly, an SVM-based model, for predicting N-linked glycosylation sites from a variety of species, including humans, mice, rats, and yeast. Wang *et al.* (2025) proposed CoNglyPred, a deep learning model that employed ESM-2 embeddings for enhanced sequence representation, while incorporating 3D structural data obtained from AlphaFold2 (Jumper *et al.* 2021) to construct protein contact graphs.

As discussed earlier, for the N-linked glycosylation to happen, it is a necessary (but not sufficient) condition that the target site must form an N-X-[S/T] sequon. Therefore, to train an N-linked glycosylation predictor, the dataset should consist only of N-X-[S/T] sequons, some of which are positive sites, and some are not. However, many earlier works used datasets consisting of sequons that did not adhere to the above motif. Recent works, such as NetNGlyc, N-GlyDE, DeepNGlyPred, LMNglyPred, EMNGly, and CoNglyPred, have on the other hand, only focused on datasets comprising N-X-[S/T] sequons and our work follows this approach as well.

A majority of these existing predictors related to N-linked glycosylation depend on input features that have been manually crafted. This dependency creates a bias toward certain characteristics and hinders the discovery of underlying representations of important but unfamiliar features. In our study, we used embedding features, obtained from various protein language models (PLMs), such as ProteinBERT (Brandes *et al.* 2022), ESM-2 (Lin *et al.* 2023), and ProtT5-XL-U50 (Elnaggar *et al.* 2022) embeddings, that are known for their wide range of successful usage in multiple protein attribute prediction tasks (Marquet *et al.* 2022, Dang and Vu 2024, Schmirler *et al.* 2024, Susanty *et al.* 2024). ProtT5-XL-U50 embeddings were utilized in (Kim and Kwon 2023, Dang and Vu 2024, Sun *et al.* 2024), ESM2 embeddings in (Gurung *et al.* 2024, Wu *et al.* 2024, Wang *et al.* 2025), and ProteinBERT embeddings in (Mishra *et al.* 2024, Simon and Bankapur 2024). The decision to incorporate these embeddings in the proposed model was inspired by their successful application in these studies. In addition to per-residue embedding, we have also utilized window representations that consider the neighboring residues for an aggregate embedding. We have also experimented with the physicochemical properties of the residues as features. To the best of our knowledge, ours is the first study to combine ProteinBERT, ESM-2, and ProtT5-XL-U50 embeddings to address the challenges of N-linked glycosylation prediction. Additionally, existing studies often overlook the importance of selecting specific feature groups for prediction. To address this, we performed incremental feature selection (IFS) on various feature groups to identify the optimal feature set for our final model. We experimented with two different datasets using 10 different machine learning (ML) classifiers and different feature groups. Per-residue as well as window features were examined. Interestingly, feature selection using IFS led to the same set of final features in both datasets. We utilized incremental mutual info (IMI) for selecting the base classifiers for our final model. Then, we did a 10-fold CV to choose the meta-classifier for our ensemble model. The learners/classifiers were further fine-tuned using grid search. Finally, we did a SHAP (SHapley Additive exPlanations) (Lundberg and Lee 2017) analysis on the selected feature set, which proved the significance of base learner probability (BLP) outputs in the stacking ensemble. As individual PLMs capture different aspects of residue-level information, our approach creates a more comprehensive feature representation by integrating multiple PLMs, which leads to improved predictive performance. Although using multiple PLMs introduces some computational cost, our model requires less expensive input data (embeddings from only protein sequences) compared to many state-of-the-art methods that need predicted or experimentally determined 3D protein structures as their input. This makes our approach more applicable to large datasets where structural information is more likely to be unavailable.

The key contributions of this paper can be summarized as follows.

- We have proposed StackGlyEmbed, a stacking ensemble method for N-linked glycosylation site prediction. StackGlyEmbed outperforms existing SOTA methods in independent datasets, achieving 98.2% sensitivity, 92.5% balanced accuracy, 89.1% F1-score, and 82.6% Matthew’s correlation coefficient (MCC).
- StackGlyEmbed utilizes multiple protein language models, such as ProtT5-XL-U50, ProteinBERT, and ESM2. To the best of our knowledge, ours is the first study that combines embeddings from multiple protein language models to predict N-linked glycosylation. We have analyzed the performance of each of the feature groups and selected our model feature set using IFS.
- In addition to using per-residue embedding, we have also used aggregate embedding across a window around the target site for the prediction task. Notably, this combined approach has been largely underexplored in recent literature on N-linked glycosylation prediction, with EMNGly being the only method that incorporates both. Combining embeddings from three PLMs in our approach provides a significant performance advantage over EMNGly, which relies on expensive structural features.
- We have analyzed the selected feature groups using SHAP (Lundberg and Lee 2017) and demonstrated the benefit of the stacking ensemble.
- We have made our proposed model freely available at https://github.com/nafcoder/StackGlyEmbed.

The rest of the sections are organized as follows. Section 2 outlines the materials and methods used in this paper. Section 3 presents the results of different experiments, performance evaluation, and comparative analysis of our approach. Section 4 explores the implications of our findings in the context of N-linked glycosylation prediction and concludes the paper with potential future directions.

## 2 Methods

**Figure 1.**
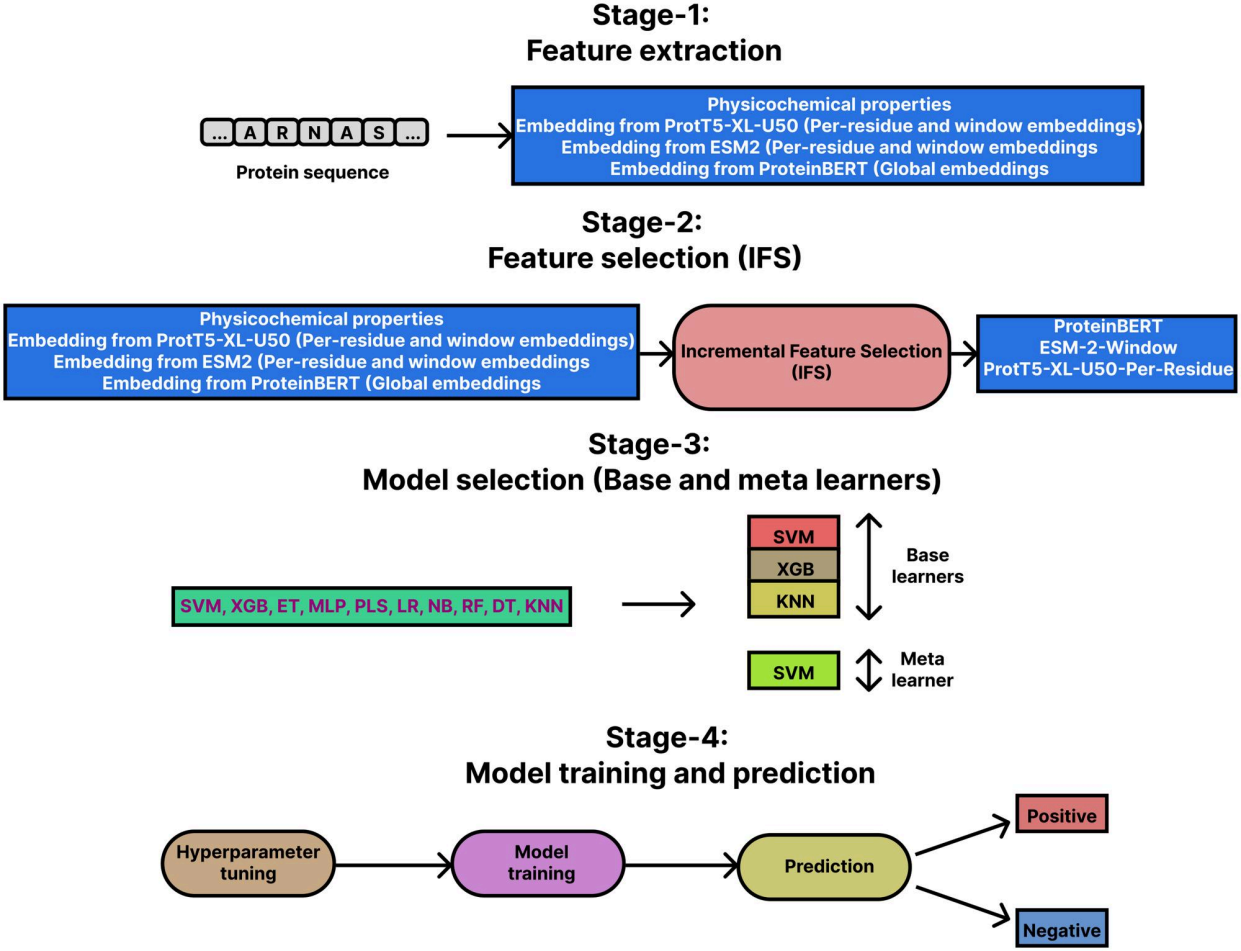
Workflow of the study consists of four stages: Feature extraction, Feature selection (IFS), Model selection (Base and meta learners), and Model training and prediction.

This section covers the datasets we have used, feature extraction and selection methods, our model framework, etc. The overall workflow is depicted in Fig. 1.

### 2.1 Dataset

In this subsection, two datasets, N-GlyDE and N-GlycositeAtlas, are described. The dataset summary is provided in Table 1.

**Table 1.** Dataset summary.

Dataset	Positive sites	Negative sites	Total sites	Proteins	Total proteins
N-GlyDE-TS	2049	1030	3079	832	918
N-GlyDE-IT	166	280	446	86	
N-GlycositeAtlas-TS	8401	15857	24258	9245	10200
N-GlycositeAtlas-IT	830	1648	2478	955	

#### 2.1.1 N-GlyDE dataset

This dataset was taken from N-GlyDE (Pitti *et al.* 2019). The authors used Uniprot (Consortium 2019) (version 201608) for preparing their datasets. From Uniprot, they collected 1068 experimentally validated N-linked human glycoproteins and 11767 non-glycoproteins. After some post-processing and ensuring a 30% sequence identity threshold using CD-HIT (Huang *et al.* 2010), 6281 proteins remained. From these, 86 proteins (comprising 53 glycoproteins and 33 non-glycoproteins), were randomly selected to prepare the independent test set. Finally, 447 N-X-[S/T] sequons were compiled from these proteins, of which 167 were glycosites (positive sites) and 280 were non-glycosites (negative sites). For training purposes, they created two datasets, referred to as the *first-stage dataset* and *second-stage dataset*. In our work, we have used the latter one as the training set. To prepare this dataset (*second-stage dataset*), the authors relaxed the sequence identity threshold to 60% and got 832 glycoproteins with a total of 3080 sequons. 2050 of them were glycosites and 1030 were non-glycosites. They also used CD-HIT to ensure that the protein sequence identity between the independent test set and the training set remains at most 30%. For a more detailed review of the data preparation protocol, the reader is referred to (Pitti *et al.* 2019). From the original datasets, we had to remove two sequons due to the corresponding protein’s primary sequence having been updated or shortened in Uniprot. In our study, the final training set (N-GlyDE-TS) thus has 2049 positive sites and 1030 negative sites, while the final independent test set (N-GlyDE-IT) has 166 positive sites and 280 negative sites (see Table 1). We further divided N-GlyDE-TS into two parts: 60% (N-GlyDE-TS-60) for training the base layer classifiers and 40% (N-GlyDE-TS-40) for validation of the base layer and training of the meta layer classifier. N-GlyDE-TS-60 contains 1229 positive and 618 negative sites, while N-GlyDE-TS-40 consists of 820 positive and 412 negative sites.

#### 2.1.2 N-GlycositeAtlas dataset

This dataset was taken from LMNglyPred (Pakhrin *et al.* 2023), where the authors took 7204 human glycoproteins from N-GlycositeAtlas (Sun *et al.* 2019). From these proteins, 9235 N-linked glycosites were obtained that were confined to N-X-[S/T] sequons. On the other hand, to construct the negative set, the authors extracted 7875 human glycoproteins from the DeepLoc-2.0 database (Thumuluri *et al.* 2022). 30% sequence identity cutoff was ensured using CD-HIT (Li and Godzik 2006). Finally, 17508 non-glycosites were obtained, confined to N-X-[S/T] sequons. From these 9235 glycosites and 17508 non-glycosites, the authors then formed a training set and an independent test set. 8405 glycosites and 15860 non-glycosites belonged to the training set while the independent test set comprised 830 glycosites and 1648 non-glycosites. For a more detailed review of the data preparation protocol, the reader is referred to (Pakhrin *et al.* 2023). From the original training set, we had to remove seven sequons due to the corresponding protein’s sequence having been updated or shortened in Uniprot, thus reducing its composition to 8401 positive and 15857 negative sites. The composition of the training set (N-GlycositeAtlas-TS) and independent test set (N-GlycositeAtlas-IT) is summarized in Table 1. Similar to the training and validation split performed in the N-GlyDE-TS, we further split N-GlycositeAtlas-TS into N-GlycositeAtlas-TS-60 (5040 positive and 9514 negative sites) and N-GlycositeAtlas-TS-40 (3361 positive and 6343 negative sites) sets.

### 2.2 Feature extraction

In this work, we have utilized physicochemical properties and embeddings from protein language models as features for the prediction problem. These are described below.

#### 2.2.1 Physicochemical properties

We used seven different physicochemical parameters (sheet probability, steric parameter, hydrophobicity, isoelectric point, normalized Van der Waals volume, helix probability and polarizability) for each of the 20 standard amino acids. We took a window of 31 residues, centered at the target site, and averaged their physicochemical parameter values. The feature group, named Physico-Window, thus had a size of 7.

#### 2.2.2 Embedding from ProtT5-XL-U50

ProtTrans (Elnaggar *et al.* 2022) has explored two auto-regressive models (Transformer-XL, XLNet) and four auto-encoder models (BERT, Albert, Electra, T5) on 393 billion amino acids of UniRef and BFD data. Among the available ProTrans models, ProtT5-XL-U50 has the highest score in CASP12, TS115, CB513 and DeepLoc for various predictions. So, we used ProtT5-XL-U50. We computed the per-residue embeddings of size 1024 and labeled them as ProtT5-XL-U50-Per-Residue. We also took a window of 31 residues, centered at the target site, and averaged their per-residue embeddings. This feature group is named ProtT5-XL-U50-Window.

#### 2.2.3 Embedding from ESM2

ESM2 (Lin *et al.* 2023) is a transformer-based protein language model that is trained using masked language modeling over millions of diverse natural proteins across evolution. It is trained for getting the inter-connected sequence patterns and their structural implications. It generates per-residue embeddings that are later used for unsupervised self-attention map contact predictions. This is then used by ESMFold (Lin *et al.* 2023) for predicting protein structures. Since we lacked the resources to investigate larger ESM2 models, we chose “esm2_t33_650M_UR50D” because it offers a balance between the model size and computational feasibility. We generated per-residue embeddings of size 1280 (ESM-2-Per-Residue) as well as embeddings averaged across a window of 31 residues (ESM-2-Window).

#### 2.2.4 Embedding from ProteinBERT

ProteinBERT (Brandes *et al.* 2022), a deep language model, was designed to capture local and global representations of proteins naturally. Its architecture consists of local and global representations that were pre-trained on around 106M proteins. Because of the efficiency and training paradigm that involves pretraining on a diverse set of biological sequences, this comparatively smaller model was explored in our study. It makes a complementary addition to our integration of PLMs due to its robustness and generalizability. We took the protein sequence and computed the per-residue, window, and global representations of it. The per-residue (ProteinBERT-Per-Residue) and window embeddings (ProteinBERT-Window) both had an embedding size of 128. The global representation (ProteinBERT) had an embedding size of 512. In Supplementary Tables S1 and S2, available as supplementary data at *Bioinformatics Advances* online, the comparisons of ProteinBERT-Per-Residue, ProteinBERT-Window, and ProteinBERT are provided on the N-GlycositeAtlas-TS and N-GlyDE-TS sets, respectively. ProteinBERT embeddings outperformed others in almost all metrics. So, we used only ProteinBERT in the rest of this study. The inferior performance of per-residue and window embeddings may be attributed to their smaller embedding size than the global embeddings. Additionally, ProteinBERT is designed to focus on global representations, as stated in (Brandes *et al.* 2022), whereas models like ProtT5-XL-U50 derive global embeddings by averaging per-residue representations.

### 2.3 Feature selection

**Figure 2.**
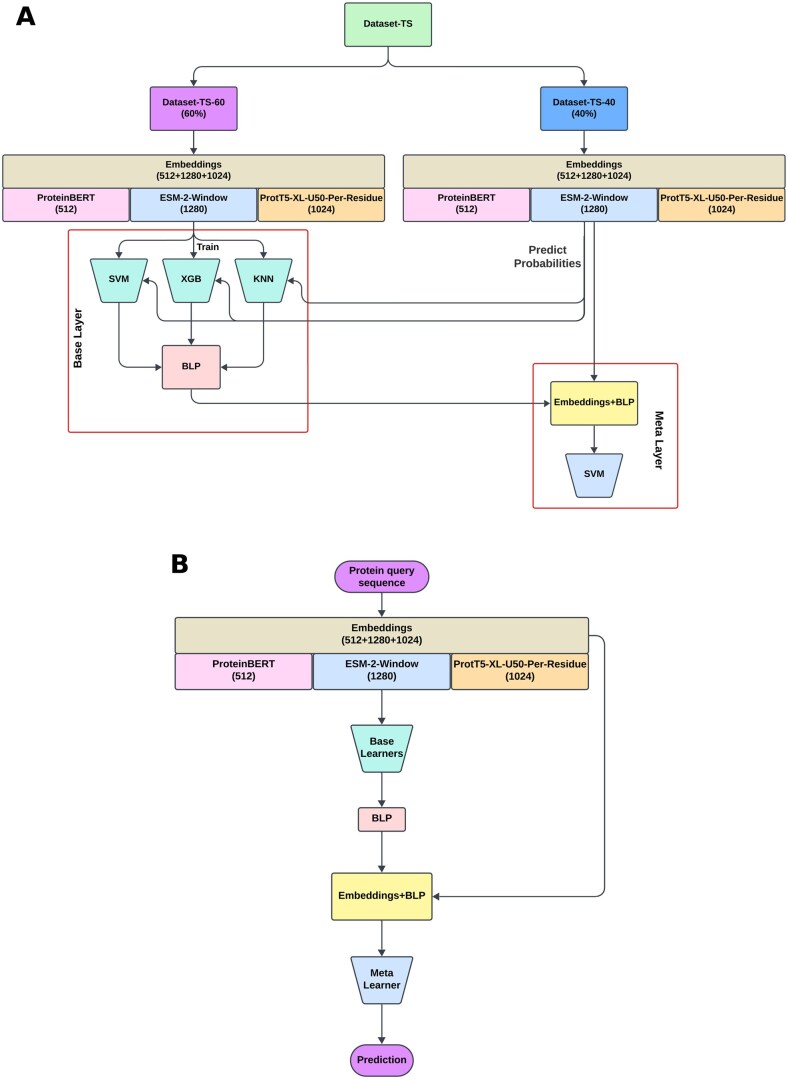
Training and prediction framework. (A) Training framework. (B) Prediction framework.

For the feature selection process, we used IFS, with SVM as the classifier. Firstly, we measured the F1-score of SVM models trained on each feature group individually and opted for the best feature group. In the next round, we augmented this feature group with each remaining feature group individually. We trained SVM models again, now on each of these feature group pairs, and measured their F1-scores. The pair that resulted in the best F1-score was kept for subsequent rounds, and we continued with this process until we reached the feature set containing all of the feature groups. Finally, among all the feature group sets thus explored, the one with the best F1-score was chosen for the final model. Accordingly, ProteinBERT, ESM-2-Window, and ProtT5-XL-U50-Per-Residue embeddings were selected as the optimal feature set. The selected feature set is shown in Fig. 2.

### 2.4 Performance evaluation

We have used various well-established metrics (Altman and Bland 1994, Fawcett 2006, Keilwagen *et al.* 2014, Powers 2020) for measuring the performance of our model. The metrics we used are balanced accuracy (BACC), accuracy (ACC), specificity (SP), sensitivity (SN), F1-score, precision (PREC), MCC, area under precision-recall curve (AUPR), and area under receiver operating characteristic curve (AUROC or AUC).


(1) $$SN=\frac{TP}{TP+FN}$$

(2) $$SP=\frac{TN}{TN+FP}$$

(3) $$\mathrm{ACC}=\frac{TP+TN}{FP+TP+TN+FN}$$

(4) $$\mathrm{BACC}=\frac{SN+SP}{2}$$

(5) $$\mathrm{PREC}=\frac{TP}{FP+TP}$$

(6) $$F1=\frac{2TP}{2TP+FP+FN}$$

(7) $$\mathrm{MCC}=\frac{\left(TP\times TN)-(FP\times FN\right)}{\sqrt{\left(TP+FN)\times (TP+FP)\times (TN+FP)\times (TN+FN\right)}}$$

Here, TP, FP, TN, FN, TPR, and FPR represent true positives, false positives, true negatives, false negatives, true positive rate, and false positive rate, respectively.

### 2.5 Summary of the framework

We constructed a stacking ensemble with two phases of learners, referred to as *base learners* in the base layer and a single *meta learner* in the meta layer, respectively. Base learners are individual machine learning models that are trained separately on the dataset, where each of the learners captures different patterns and relationships. Meta learner is the model that takes both the predictions from the base learners and the initial input features as inputs. By learning from both sources, the meta learner enhances the final prediction. Two separate ensembles were trained on the two datasets, N-GlyDE and N-GlycositeAtlas, following an identical methodology. Therefore, we describe the process for the N-GlyDE dataset only. Notably, the data was pre-processed using Yeo–Johnson transformation (Yeo and Johnson 2000) to make it more Gaussian-like. We used the N-GlyDE-TS-60 set to train the base learners, while N-GlyDE-TS-40 was used to train the meta learner. The data was featurized using the optimal feature set determined by the IFS method described earlier. When training the meta layer, the feature vectors for N-GlyDE-TS-40 were augmented using the probabilities obtained from the base layer, referred to as the BLP vector.

The selection of base learners was made using the Incremental Mutual Information (IMI) approach that utilized Mutual Information (MI) (Vergara and Estévez 2014) between learner outputs and the actual labels. IMI works similarly to the IFS method. The only differences are that IMI is searching for an optimal set of learners (instead of an optimal feature set) and the objective function here is mutual information (instead of F1-score). We analyzed 10 classifiers in total which are: Support Vector Machine (SVM), Extreme Gradient Boosting (XGB), Extra Tree (ET), Multi-layer Perceptron (MLP), Partial Least Square Regression (PLS), Logistic Regression (LR), Naive Bayes (NB), Random Forest (RF), Decision Tree (DT) and *K*-nearest Neighbor (KNN). IMI resulted in the selection of SVM, XGB, and KNN as the base learners. On the other hand, SVM was chosen as the meta learner since it was the best individual classifier in terms of predictive performance (see Results). The overall framework for training and inference is shown in Fig. 2.

### 2.6 Resolving data imbalance

There is a notable imbalance in the datasets used in this research. For N-GlyDE the positive sites are almost double the number of the negative sites while it is the other way around for N-GlycositeAtlas. Inspired by (Xia *et al.* 2020), we followed an ensemble random undersampling strategy for the N-GlyDE-TS-60 and N-GlycositeAtlas-TS-60 datasets to train the base learners. We describe the process for N-GlyDE-TS-60 only. Ten subsampled datasets were created from it, including all sites of the minority class and varying percentages of sites from the majority class—three samples contained 70% of the majority class, four contained 50%, and three contained 30%. The three base learners (SVM, XGB, and KNN) were trained on these 10 datasets, thus rendering thirty trained models in the base layer. For the meta learner training, on the other hand, the N-GlyDE-TS-40 and N-GlycositeAtlas-TS-40 datasets were balanced using random undersampling.

## 3 Results

### 3.1 ProteinBERT, ESM-2-Window and ProtT5-XL-U50-Per-Residue constitute the optimal feature set

**Figure 3.**
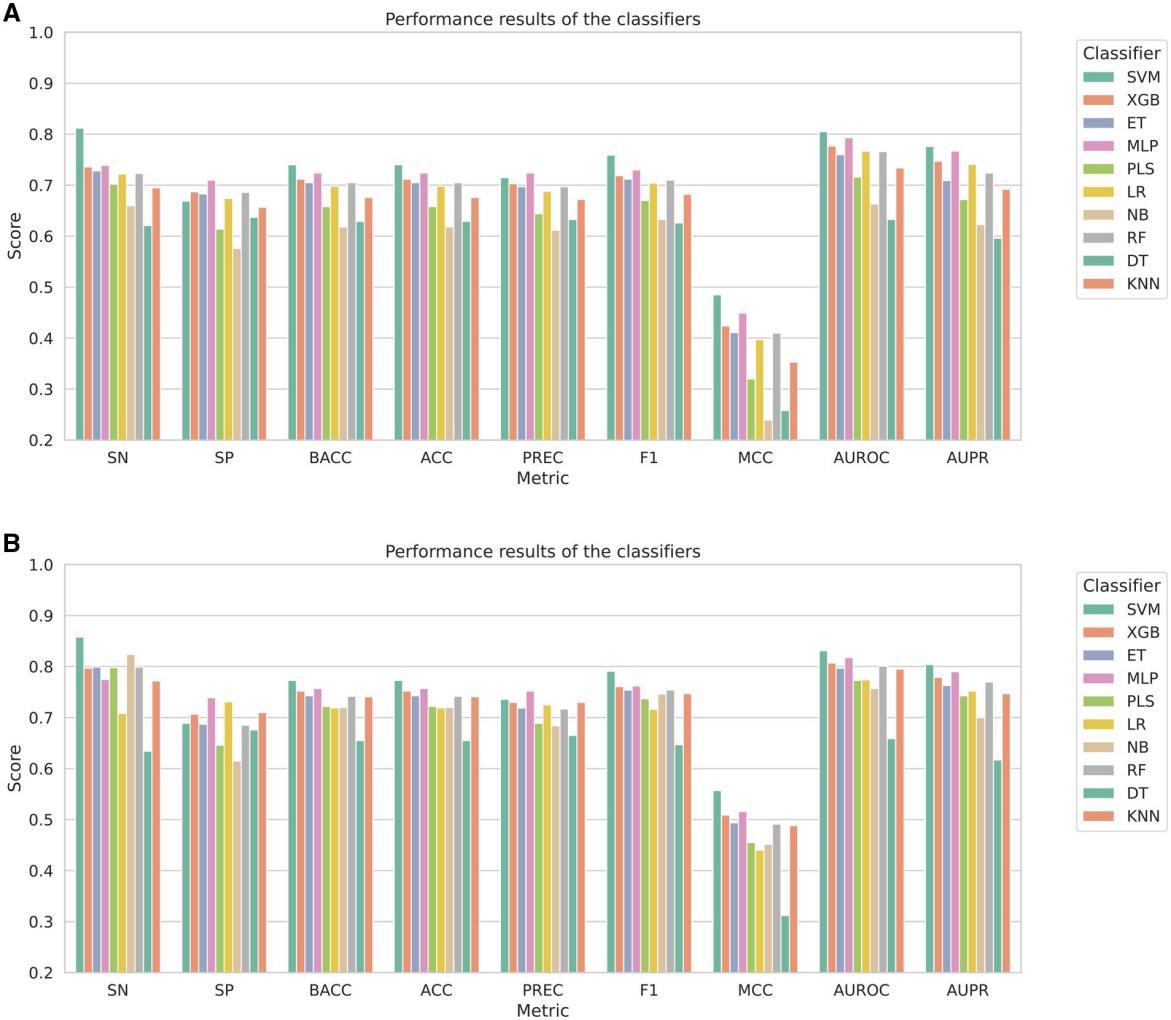
Comparison of classifier performances across two datasets: N-GlycositeAtlas-TS and N-GlyDE-TS sets. (A) 10-Fold CV performance of all 10 classifiers, averaged over all 6 feature groups, trained on the N-GlycositeAtlas-TS set that was balanced using random undersampling. (B) 10-Fold CV performance of all 10 classifiers, averaged over all 6 feature groups, trained on the N-GlyDE-TS set that was balanced using random undersampling.

As mentioned in Section 2.3, we used IFS with SVM for feature selection. The choice of SVM here was not arbitrary. Rather, each of the 10 ML classifiers was trained separately on each of the 6 feature groups. For each classifier, we illustrated various performance metrics, averaged across the feature groups, in Fig. 3A and B, for the two different training datasets, respectively. In both cases, SVM achieved the best performance based on F1, MCC, AUROC, and AUPR scores, which justified its incorporation with the IFS method for feature selection.

The results of IFS are recorded in Tables 2 (for N-GlycositeAtlas) and 3 (for N-GlyDE). In both cases, (ProteinBERT, ESM-2-Window, ProtT5-XL-U50-Per-Residue) feature set resulted in the highest F1-score. Therefore, it was selected as the optimal feature set. The length of the final feature vector is 2816, with each individual feature group’s feature count shown in Fig. 2. For ease of subsequent reference, we have named this feature combination as FC-1. When augmented with BLP, we refer to it as FC-2 (see Table 7).

**Table 2.** 10-Fold CV performance of SVM classifier, trained on N-GlycositeAtlas-TS, using IFS.^a^

Feature set	SN	SP	BACC	ACC	PREC	F1	MCC	AUROC	AUPR
Physico-Window	0.721	0.436	0.579	0.579	0.561	0.631	0.164	0.607	0.579
ProtT5-XL-U50-Per-Residue	0.839	0.686	0.763	0.763	0.728	0.779	0.532	0.834	0.802
ProtT5-XL-U50-Window	0.848	0.703	0.775	0.775	0.741	0.79	0.556	0.842	0.806
ESM-2-Per-Residue	0.806	0.682	0.744	0.744	0.717	0.759	0.492	0.814	0.78
ESM-2-Window	0.832	0.715	0.774	0.774	0.745	0.786	0.552	0.848	0.819
ProteinBERT	0.825	0.789	0.807	0.807	0.797	0.811	0.615	0.884	0.87
ProteinBERT, Physico-Window	0.827	0.786	0.807	0.807	0.795	0.81	0.614	0.886	0.874
ProteinBERT, ProtT5-XL-U50-Per-Residue	0.854	0.742	0.798	0.798	0.768	0.809	0.6	0.871	0.851
ProteinBERT, ProtT5-XL-U50-Window	0.856	0.743	0.799	0.799	0.769	0.81	0.603	0.871	0.846
ProteinBERT, ESM-2-Per-Residue	0.833	0.73	0.782	0.782	0.756	0.792	0.567	0.854	0.83
ProteinBERT, ESM-2-Window	0.851	0.753	0.802	0.802	0.775	0.811	0.607	0.875	0.855
ProteinBERT, ESM-2-Window, Physico-Window	0.851	0.753	0.802	0.802	0.775	0.811	0.607	0.875	0.855
**ProteinBERT, ESM-2-Window, ProtT5-XL-U50-Per-Residue**	**0.857**	**0.749**	**0.803**	**0.803**	**0.774**	**0.813**	**0.61**	**0.877**	**0.858**
ProteinBERT, ESM-2-Window, ProtT5-XL-U50-Window	0.86	0.741	0.8	0.8	0.769	0.811	0.605	0.873	0.849
ProteinBERT, ESM-2-Window, ESM-2-Per-Residue	0.839	0.739	0.789	0.789	0.763	0.799	0.581	0.863	0.842
ProteinBERT, ESM-2-Window, ProtT5-XL-U50-Per-Residue, Physico-Window	0.856	0.749	0.803	0.803	0.774	0.813	0.609	0.877	0.858
ProteinBERT, ESM-2-Window, ProtT5-XL-U50-Per-Residue, ProtT5-XL-U50-Window	0.857	0.744	0.8	0.8	0.77	0.811	0.605	0.875	0.855
ProteinBERT, ESM-2-Window, ProtT5-XL-U50-Per-Residue, ESM-2-Per-Residue	0.849	0.738	0.794	0.794	0.765	0.804	0.591	0.867	0.844
ProteinBERT, ESM-2-Window, ProtT5-XL-U50-Per-Residue, Physico-Window, ProtT5-XL-U50-Window	0.857	0.744	0.8	0.8	0.77	0.811	0.604	0.875	0.855
ProteinBERT, ESM-2-Window, ProtT5-XL-U50-Per-Residue, Physico-Window, ESM-2-Per-Residue	0.848	0.739	0.793	0.793	0.765	0.804	0.591	0.867	0.845
ProteinBERT, ESM-2-Window, ProtT5-XL-U50-Per-Residue, Physico-Window, ProtT5-XL-U50-Window, ESM-2-Per-Residue	0.853	0.737	0.795	0.795	0.764	0.806	0.594	0.869	0.847

a Data was balanced using random undersampling. The feature combination with the highest F1-score is chosen (shown in boldface).

**Table 3.** 10-Fold CV performance of SVM classifier, trained on N-GlyDE-TS, using IFS.^a^

Feature set	SN	SP	BACC	ACC	PREC	F1	MCC	AUROC	AUPR
Physico-Window	0.725	0.496	0.611	0.611	0.59	0.651	0.228	0.648	0.628
ProtT5-XL-U50-Per-Residue	0.93	0.712	0.821	0.821	0.764	0.839	0.658	0.894	0.87
ProtT5-XL-U50-Window	0.908	0.733	0.82	0.82	0.773	0.835	0.651	0.887	0.854
ESM-2-Per-Residue	0.924	0.692	0.808	0.808	0.752	0.829	0.635	0.87	0.834
ESM-2-Window	0.92	0.746	0.833	0.833	0.785	0.847	0.677	0.896	0.865
ProteinBERT	0.739	0.754	0.747	0.747	0.751	0.744	0.494	0.792	0.773
ESM-2-Window, Physico-Window	0.918	0.745	0.832	0.832	0.784	0.845	0.674	0.896	0.865
ESM-2-Window, ProtT5-XL-U50-Per-Residue	0.931	0.752	0.842	0.842	0.791	0.855	0.695	0.91	0.886
ESM-2-Window, ProtT5-XL-U50-Window	0.926	0.734	0.83	0.83	0.777	0.845	0.673	0.899	0.869
ESM-2-Window, ESM-2-Per-Residue	0.924	0.746	0.835	0.835	0.786	0.849	0.683	0.897	0.867
ESM-2-Window, ProteinBERT	0.912	0.776	0.844	0.844	0.804	0.854	0.695	0.909	0.883
ESM-2-Window, ProtT5-XL-U50-Per-Residue, Physico-Window	0.931	0.752	0.842	0.842	0.791	0.855	0.695	0.91	0.886
ESM-2-Window, ProtT5-XL-U50-Per-Residue, ProtT5-XL-U50-Window	0.933	0.753	0.843	0.843	0.792	0.856	0.698	0.913	0.89
ESM-2-Window, ProtT5-XL-U50-Per-Residue, ESM-2-Per-Residue	0.934	0.741	0.837	0.837	0.784	0.852	0.689	0.904	0.879
**ESM-2-Window, ProtT5-XL-U50-Per-Residue, ProteinBERT**	**0.932**	**0.779**	**0.855**	**0.855**	**0.81**	**0.866**	**0.72**	**0.927**	**0.908**
ESM-2-Window, ProtT5-XL-U50-Per-Residue, ProteinBERT, Physico-Window	0.932	0.779	0.855	0.855	0.81	0.866	0.72	0.927	0.908
ESM-2-Window, ProtT5-XL-U50-Per-Residue, ProteinBERT, ProtT5-XL-U50-Window	0.93	0.772	0.851	0.851	0.804	0.862	0.711	0.924	0.905
ESM-2-Window, ProtT5-XL-U50-Per-Residue, ProteinBERT, ESM-2-Per-Residue	0.934	0.77	0.852	0.852	0.803	0.864	0.714	0.92	0.898
ESM-2-Window, ProtT5-XL-U50-Per-Residue, ProteinBERT, Physico-Window, ProtT5-XL-U50-Window	0.93	0.773	0.851	0.851	0.805	0.863	0.712	0.924	0.904
ESM-2-Window, ProtT5-XL-U50-Per-Residue, ProteinBERT, Physico-Window, ESM-2-Per-Residue	0.934	0.769	0.851	0.851	0.803	0.863	0.713	0.919	0.898
ESM-2-Window, ProtT5-XL-U50-Per-Residue, ProteinBERT, Physico-Window, ESM-2-Per-Residue, ProtT5-XL-U50-Window	0.931	0.769	0.85	0.85	0.802	0.862	0.71	0.919	0.897

a Data was balanced using random under sampling. The feature combination with the highest F1-score is chosen. In case of a tie, the combination with a lesser number of feature groups is selected (shown in boldface).

**Table 7.** Feature combinations.

Feature combination label	Feature set
FC-1	ProteinBERT, ESM-2-Window, ProtT5-XL-U50-Per-Residue
FC-2	ProteinBERT, ESM-2-Window, ProtT5-XL-U50-Per-Residue, BLP

### 3.2 Choice of learners in base and meta layers

The set of classifiers having the highest MI score on each round using Incremental Mutual Information (IMI) is shown in Tables 4 (for N-GlycositeAtlas) and 5 (for N-GlyDE). Full tables with performance metrics are given in Supplementary Material, available as supplementary data at *Bioinformatics Advances* online. In both cases, the combination of SVM, XGB, and KNN produced the highest MI score. Therefore, these classifiers were chosen as the base learners.

**Table 4.** The highest MI scores of the learner combinations in each round.^a^

Learner Combination	MI
SVM	0.2241
SVM, XGB	0.2345
**SVM**, **XGB**, **KNN**	**0.239**
SVM, XGB, KNN, PLS	0.2361
SVM, XGB, KNN, PLS, MLP	0.2358
SVM, XGB, KNN, PLS, MLP, RF	0.2376
SVM, XGB, KNN, PLS, MLP, RF, LR	0.2317
SVM, XGB, KNN, PLS, MLP, RF, LR, ET	0.2239
SVM, XGB, KNN, PLS, MLP, RF, LR, ET, NB	0.2178
SVM, XGB, KNN, PLS, MLP, RF, LR, ET, NB, DT	0.2066

a The learners were trained on the randomly undersampled N-GlycositeAtlas-TS-60 set, and MI scores were measured on the N-GlycositeAtlas-TS-40 set. The learner combination with the highest MI score is chosen (shown in boldface).

**Table 5.** Highest MI scores of the learner combinations in each round.^a^

Learner Combination	MI
XGB	0.3316
XGB, KNN	0.3425
**XGB**, **KNN**, **SVM**	**0.3664**
XGB, KNN, SVM, NB	0.3572
XGB, KNN, SVM, NB, ET	0.3518
XGB, KNN, SVM, NB, ET, MLP	0.3498
XGB, KNN, SVM, NB, ET, MLP, LR	0.3522
XGB, KNN, SVM, NB, ET, MLP, LR, DT	0.353
XGB, KNN, SVM, NB, ET, MLP, LR, DT, PLS	0.3422
XGB, KNN, SVM, NB, ET, MLP, LR, DT, RF, PLS	0.3299

a The learners were trained on the randomly undersampled N-GlyDE-TS-60 set and MI scores were measured on the N-GlyDE-TS-40 set. The learner combination with the highest MI score is chosen (shown in boldface).

**Figure 4.**
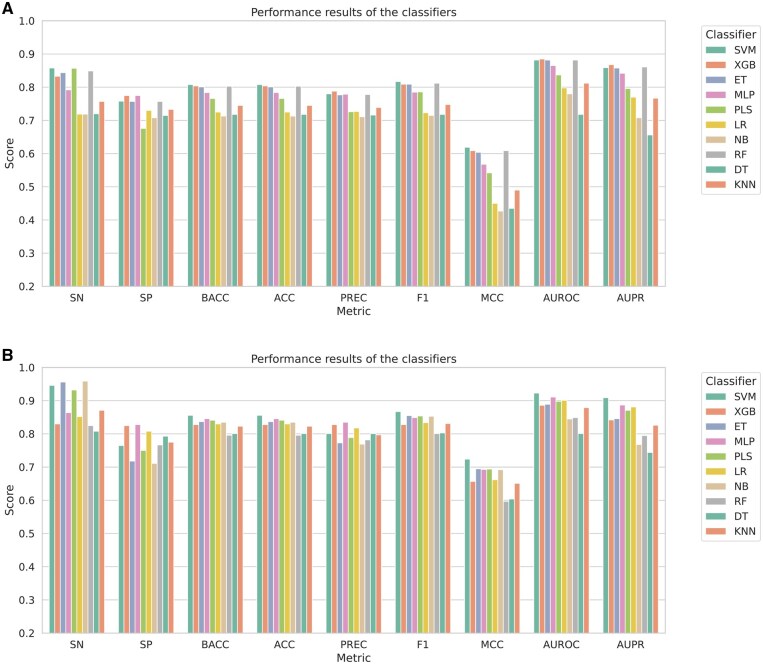
Comparison of classifier performances across two datasets: N-GlycositeAtlas-TS-40 and N-GlyDE-TS-40 sets. (A) 10-Fold CV performance of 10 ML models on N-GlycositeAtlas-TS-40, balanced with random undersampling. The feature set was augmented using BLP. (B) 10-Fold CV performance of 10 ML models on N-GlyDE-TS-40, balanced with random undersampling. The feature set was augmented using BLP.

After settling on the base learners, we experimented with the potential meta learners as follows. We measured the 10-Fold CV performance of 10 ML models on the randomly undersampled N-GlycositeAtlas-TS-40 set and N-GlyDE-TS-40 set and illustrated them in Fig. 4A and B, respectively. Notably, the feature set was augmented using BLP. In both datasets, SVM outperformed the other learners. Therefore, we selected it as the meta learner of our stacking ensemble.

### 3.3 Hyperparameter tuning

We used Grid Search to tune the hyperparameters of the different classifiers. The searched grids and the finally selected values for each classifier are reported in Table 6. The full table of each learner with their performances at the different parameter combinations is given in Supplementary Material, available as supplementary data at *Bioinformatics Advances* online. We also chose the window size as a hyperparameter and conducted experiments with different window sizes that are provided in Supplementary Tables S3 and S4, available as supplementary data at *Bioinformatics Advances* online. We strategically chose various window sizes to cover short and relatively longer-range dependencies. Specifically, we analyzed the following window sizes: 21, 27, 31, 37, and 41. In Supplementary Table S4, available as supplementary data at *Bioinformatics Advances* online, the window size of 31 outperformed other window sizes. In Supplementary Table S3, available as supplementary data at *Bioinformatics Advances* online, the performance of window sizes other than 31 showed minimal differences in metrics compared to 31. However, the window size of 31 achieved the highest AUROC and AUC scores. Based on these results, we selected 31 as the final window size.

**Table 6.** List of learner hyperparameters and their final (tuned) values.

Layers	Classifiers	Attributes	Searched values	Tuned Value
Base layer	SVM	degree	3, 5	3
		kernel	linear, poly, rbf, sigmoid	rbf
		gamma	scale, auto	scale
		C	0.01, 0.1, 1, 10	1
	XGB	eta	0.001, 0.3	0.3
		max_depth	6, 10	10
		subsample	0.6, 1.0	1.0
		colsample_bytree	0.6, 1.0	0.6
		gamma	0, 5	0
		alpha	0, 0.1	0
		lambda	0, 0.5	0.5
	KNN	n_neighbors	5, 15, 30	30
		weights	uniform, distance	distance
		algorithm	auto, ball_tree, kd_tree	auto
		leaf_size	20, 30, 50	20
		p	1, 2	1
Meta layer	SVM	degree	3, 5	3
		kernel	linear, poly, rbf, sigmoid	rbf
		gamma	scale, auto	scale
		C	0.01, 0.1, 1, 10	1

### 3.4 Significance of BLP on the performance

**Figure 5.**
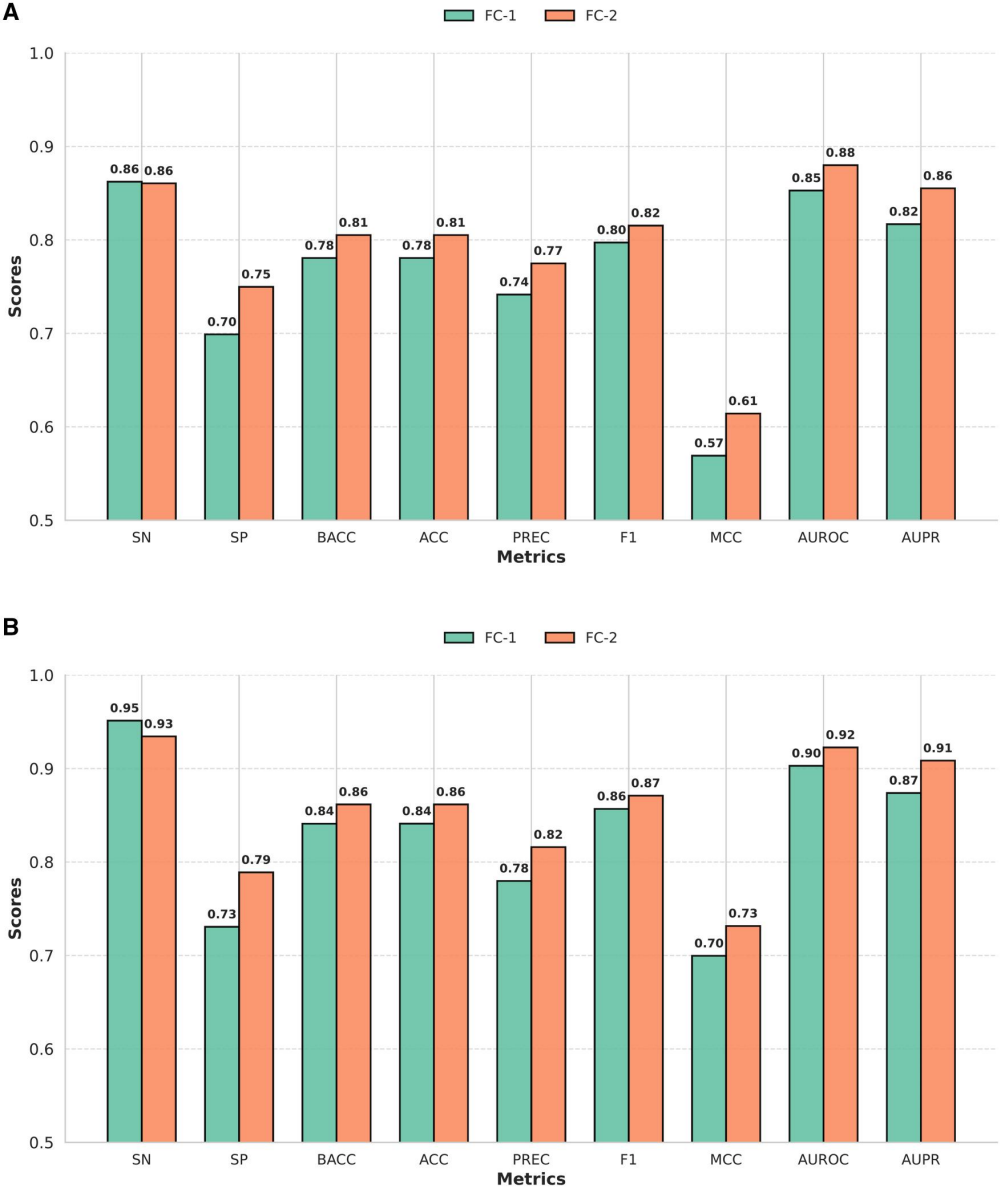
Performance comparison of meta classifiers without BLP (FC-1) and with BLP (FC-2) on the N-GlycositeAtlas-TS-40 and N-GlyDE-TS-40 sets. (A) 10-Fold CV performance of meta classifier without BLP (FC-1) and with BLP (FC-2) on the randomly undersampled N-GlycositeAtlas-TS-40 set. (B) 10-Fold CV performance of meta classifier without BLP (FC-1) and with BLP (FC-2) on the randomly undersampled N-GlyDE-TS-40 set.

To examine the benefit of the BLP being added to the feature vector before it is passed through the meta layer of the stacking ensemble, we compared the performance of the meta classifier without and with the BLP. These two variations of feature vectors are referred to as FC-1 and FC-2, respectively (Table 7). The results are shown in Fig. 5A and B. In both cases, we can see that FC-2 performs better than FC-1 in terms of almost all metrics. Thus, the contribution of BLP in improving the efficacy of the model is evident. As base learner probabilities are derived from the base learners and not influenced by the meta learner, this comparison evaluates the added value of the meta learner beyond what is already captured by the base learners. It ensures that the performance gains are not artificially inflated by reusing the same information in both layers.

**Figure 6.**
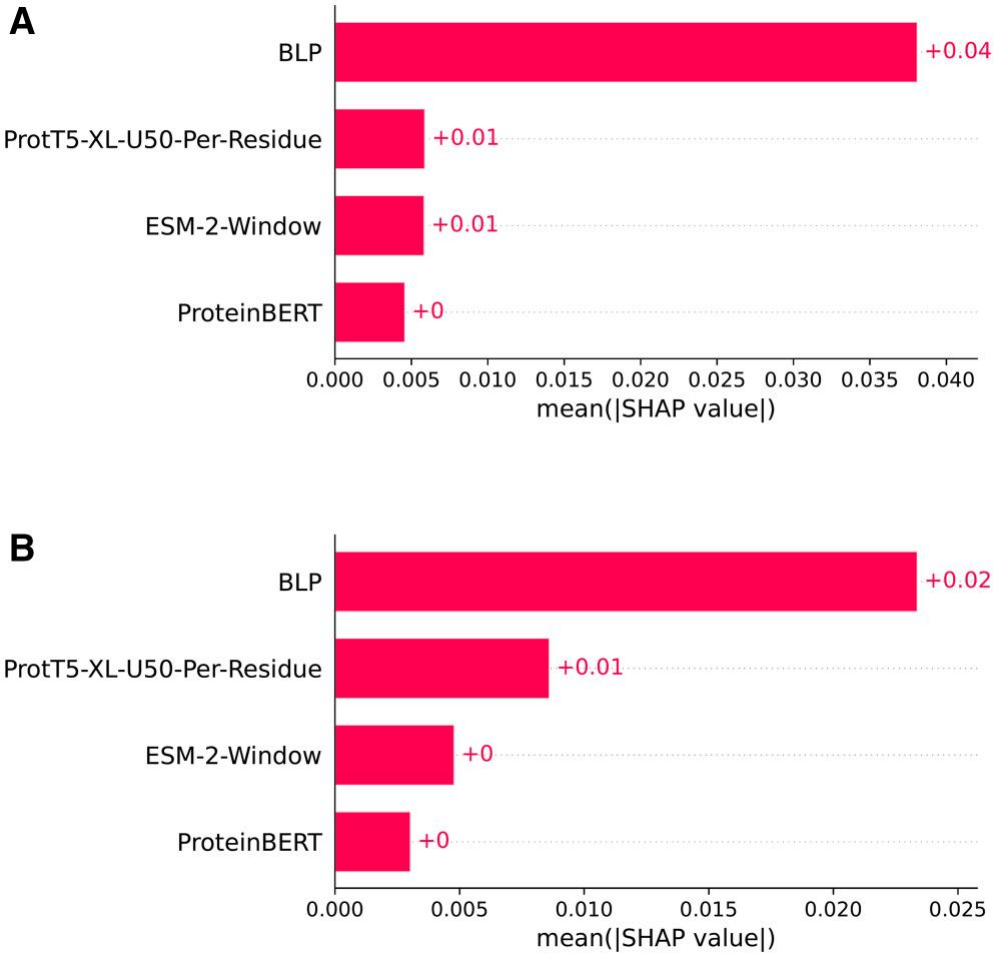
SHAP analysis on the N-GlycositeAtlas-TS-40 and N-GlyDE-TS-40 datasets. (A) Bar plot of mean absolute SHAP values of each feature group across samples of the N-GlycositeAtlas-TS-40 dataset. (B) Bar plot of mean absolute SHAP values of each feature group across samples of the N-GlyDE-TS-40 dataset.

To further examine the contribution of BLP as well as the other feature groups, we performed SHAP (Lundberg and Lee 2017) analysis as follows. The dimension of our feature vector is 2846 with BLP (1024 from ProtT5-XL-U50-Per-Residue, 1280 from ESM-2-Window, 512 from ProteinBERT and 30 from BLP). As it is prohibitively expensive to conduct SHAP analysis with large feature vectors, we reduced the feature dimension to 100 using PCA (Principal component analysis) (Abdi and Williams 2010). We applied PCA independently on each feature group and collected 10 principal components (PCs) from the BLP vector and 30 PCs each from the other three feature groups. These were then concatenated to derive the 100-dimensional reduced feature space. As the computation time of SHAP also increases considerably with sample size, we used sampling in the N-GlycositeAtlas-TS-40 dataset to choose 1078 samples, comprising equal number of positive and negative samples. As the N-GlyDE-TS-40 dataset was relatively small, we only used random undersampling to balance it, which resulted in a total of 824 samples. SHAP analysis was then performed and SHAP values were obtained for each feature of each sample. Using these, the mean absolute SHAP values were computed for each feature group, which are shown in the bar plots of Fig. 6A (for N-GlycositeAtlas-TS-40 dataset) and B (for N-GlyDE-TS-40 dataset). In both cases, BLP has a considerably higher mean absolute SHAP value compared to the other feature groups. This indicates the efficacy of BLP in discriminating between the positive and negative classes.

### 3.5 Comparison with the state-of-the-art methods

Performance of different state-of-the-art (SOTA) methods has been compared with StackGlyEmbed on the N-GlycositeAtlas-IT and the N-GlyDE-IT datasets, respectively, in Tables 8 and 9. In both cases, StackGlyEmbed outperformed SOTA methods in terms of SN, F1, MCC, AUROC, and AUPR. When trained on the N-GlycositeAtlas-TS dataset, it has 8.63% more sensitivity, 2.06% more F1-score, and 3.83% more MCC than LMNglyPred on the N-GlycositeAtlas-IT dataset. On the other hand, StackGlyEmbed trained on N-GlyDE-TS has 31.46% more sensitivity, 10% more F1-score, and 15.04% more MCC than LMNglyPred, and has 0.72% more sensitivity and 12.23% more MCC than EMNGly on N-GlyDE-IT dataset. Our model significantly increased the sensitivity on the N-GlyDE-IT dataset compared to the SOTA methods.

**Table 8.** Comparison of StackGlyEmbed with SOTA methods on the N-GlycositeAtlas-IT independent dataset.

Predictor	SN	SP	BACC	ACC	PREC	F1	MCC	AUROC	AUPR
StackGlyEmbed	**0.831**	0.714	**0.772**	0.753	0.594	**0.693**	**0.515**	**0.836**	**0.659**
LMNglyPred	0.765	**0.754**	0.759	**0.757**	**0.61**	0.679	0.496	0.759	0.545

The boldfaced values indicate the best performance for each evaluation metric.

**Table 9.** Comparison of StackGlyEmbed with SOTA methods on the N-GlyDE-IT independent dataset.

Predictor	SN	SP	BACC	ACC	PREC	F1	MCC	AUROC	AUPR
StackGlyEmbed	**0.982**	0.868	**0.925**	**0.91**	0.815	**0.891**	**0.826**	**0.967**	**0.931**
LMNglyPred	0.747	**0.942**	0.845	0.869	**0.886**	0.81	0.718	0.845	0.756
DeepNGlyPred	0.725	0.811	0.768	0.779	0.695	0.71	0.531	0.768	0.607
EMNGly^a^	0.975	0.7		0.884			0.736	0.946	
N-GlyDE^b^	0.826	0.689		0.74	0.613		0.499		
GlycoMine^b^	0.70	0.739		0.725	0.616		0.43		
NetNGlyc^b^	0.844	0.411		0.572	0.46		0.265		
GlycoEP_Std_PPP^b^	0.512	0.61		0.574	0.437		0.119		

a Taken from (Hou *et al.* 2023).

b Taken from (Pakhrin *et al.* 2023).

The boldfaced values indicate the best performance for each evaluation metric.

For the N-GlycositeAtlas dataset, we could only consider LMNglyPred as the comparative method. There are three other SOTA methods, namely DeepNGlyPred, EMNGly, and CoNglyPred, which have also utilized the N-GlycositeAtlas dataset. However, DeepNGlyPred used the entire N-GlycositeAtlas dataset to train their model. Therefore, N-GlycositeAtlas-IT cannot be used to independently assess its performance for a comparative analysis. EMNGly, on the other hand, augmented the N-GlycositeAtlas dataset (both the training and testing parts) with data from several other sources. Therefore, performance reports from their paper cannot be used for comparison. Instead, we have to use the EMNGly model for inference on N-GlycositeAtlas-IT and calculate the various metrics. However, the GitHub repository of EMNGly did not contain the required features for training and making predictions. The required feature CSV files were not available, making it non-reproducible. Authors of CoNglyPred curated their own dataset from the UniProt/Swiss-Prot database (Consortium 2019) and N-GlycositeAtlas database (Sun *et al.* 2019). Therefore, we need to use the CoNglyPred model for inference on N-GlycositeAtlas-IT and compute various metrics. However, CoNglyPred requires AlphaFold2 structural PDB files as input, which are computationally expensive to generate.

**Figure 7.**
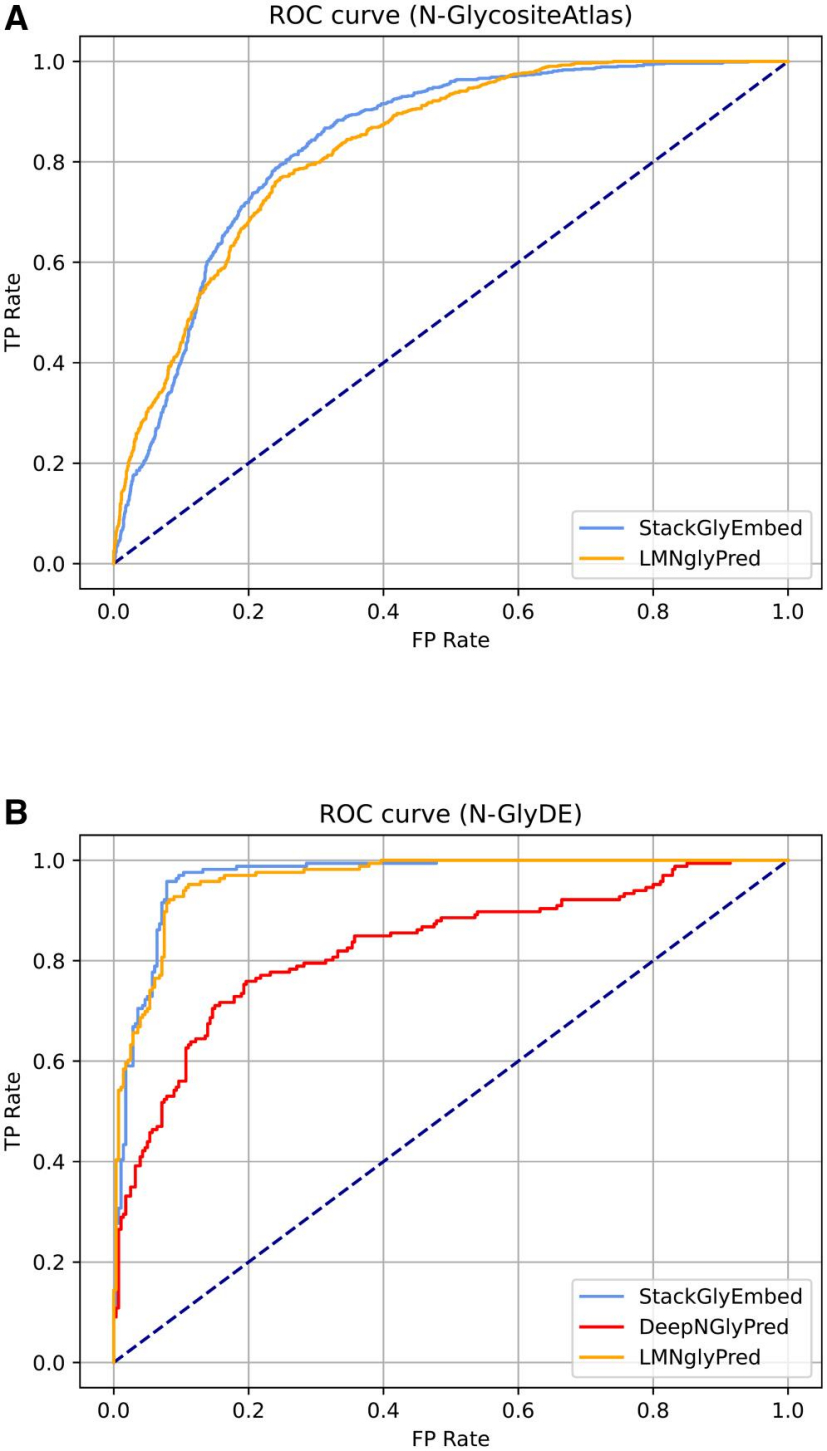
Comparison of ROC curves of StackGlyEmbed and SOTA methods on the independent sets with models trained on the corresponding training sets. (A) ROC curve for N-GlycositeAtlas-IT independent dataset. (B) ROC curve for N-GlyDE-IT independent dataset.

The ROC (Receiver Characteristic curve) curves of StackGlyEmbed and LMNglyPred in N-GlycositeAtlas, and StackGlyEmbed, LMNglyPred and DeepNGlyPred in N-GlyDE are plotted in Fig. 7. Our model significantly performs better than the other existing models. Notably, the models perform worse when trained on N-GlycositeAtlas-TS vs. on N-GlyDE-TS. The authors of LMNglyPred reasoned that the N-GlycositeAtlas dataset is not properly validated and a probable cause is the possibility of spontaneous deamidation (Palmisano *et al.* 2012). Accordingly, StackGlyEmbed trained on the N-GlyDE-TS dataset has been determined as our final prediction model and our GitHub scripts use this model for inference tasks.

Additionally, we also trained a feed-forward neural network (FNN) with our optimal set of feature groups on the N-GlycositeAtlas-TS and N-GlyDE-TS sets and respectively tested on N-GlycositeAtlas-IT and N-GlyDE-IT. The FNN delivered subpar performance compared to StackGlyEmbed, achieving 0.751 BACC, 0.669 F1, and 0.482 MCC on the N-GlycositeAtlas-IS set, and 0.894 BACC, 0.866 F1, and 0.785 MCC on the N-GlyDE-IS set. This highlights the effectiveness of using a stacking ensemble of traditional ML models over deep learning models for this task.

We did a cross-dataset evaluation by training on one dataset and testing on the other to assess the generalizability of the method, and the results are provided in Supplementary Tables S5 and S6, available as supplementary data at *Bioinformatics Advances* online. Unfortunately, the performance was poor in both cases. Notably, even the only state-of-the-art method that had the necessary trained models on their GitHub and had the same dataset settings for both datasets, LMNglyPred, performed poorly under these conditions. It demonstrates the challenge of cross-dataset prediction in this task and suggests the presence of a domain adaptation problem, where data distribution or sequence characteristics between the N-GlyDE and N-GlycositeAtlas datasets hinder model transferability. The models trained on one dataset fail to do well on the other. A probable reason may be dataset-specific biases that impact feature representations. To further analyze this issue, we created merged non-redundant training and test sets by merging the N-GlyDE-TS and N-GlycositeAtlas-TS training sets, as well as the N-GlyDE-IT and N-GlycositeAtlas-IT test sets, followed by redundancy reduction using CD-HIT at a 30% sequence similarity threshold. The results on these merged datasets are provided in Supplementary Table S7, available as supplementary data at *Bioinformatics Advances* online. The results illustrate significant performance improvement. We could not compare these results with LMNglyPred because the trained model of LMNglyPred on the newly created merged dataset was not available. The results suggest that integrating data from both sources helps the model learn more generalizable patterns that are less sensitive to dataset-specific biases. These findings highlight that domain adaptation remains a challenge in this task, and future studies can explore different techniques to bridge the gap between these two datasets.

### 3.6 Case study

**Figure 8.**
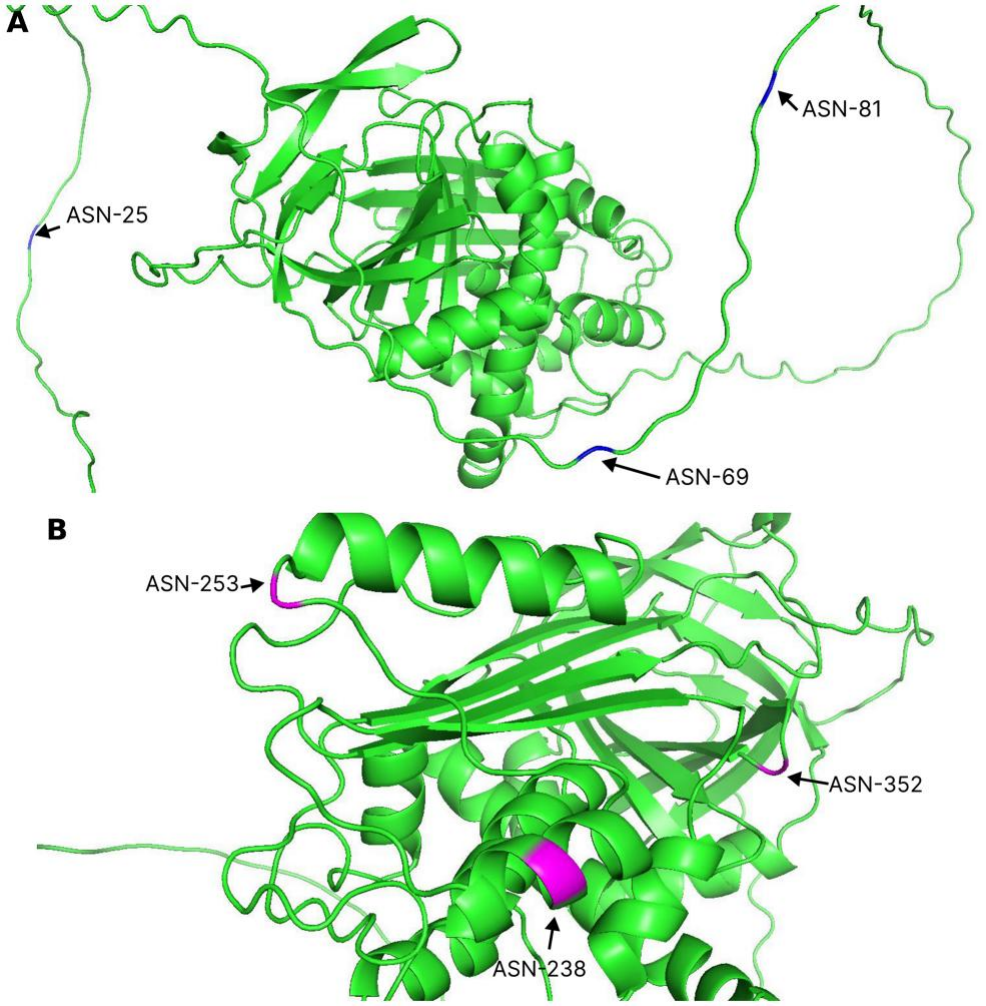
Case study of the protein with UniProt ID P05155. (A) Asparagine (ASN) sites were correctly predicted by StackGlyEmbed but missed by LMNglyPred and DeepNGlyPred. The identified residue positions—25, 69, and 81—are shown in blue and located within thin wavy lines, representing loops or random coils. These regions are not part of defined secondary structure elements like helices or sheets, but instead are flexible connecting regions. (B) Asparagine (ASN) sites correctly predicted by StackGlyEmbed, LMNglyPred, and DeepNGlyPred. The identified residue positions—238, 253, and 352—are shown in magenta and are located within or near alpha helices (spiral coils) or beta sheets (flat arrows), which represent defined secondary structure elements.

We did a case study on the protein with UniProt ID P05155. We collected the protein structure predicted by AlphaFold2 and visualized it using PyMOL (DeLano *et al.* 2002). This protein contains six N-linked glycosylation sites at positions 25, 69, 81, 238, 253, and 352. Among these, StackGlyEmbed correctly predicted all glycosylation sites, whereas LMNglyPred and DeepNGlyPred failed to identify the first three of them: 25, 69, and 81. As shown in Fig. 8, residues at positions 25, 69, and 81 are located within thin wavy lines. These wavy lines indicate loops or random coils—flexible connecting regions not part of defined secondary structure elements like alpha helices or beta sheets. In contrast, residues 238, 253, and 352 are situated within or near alpha helices (spiral coils) or beta sheets (flat arrows), which are secondary structured regions. This suggests that the other two state-of-the-art methods struggled to predict sites within flexible regions. StackGlyEmbed was successful in these regions likely due to the fusion of diverse PLM embeddings better capturing sequence-level and contextual patterns.

## 4 Discussion

In this paper, we have proposed StackGlyEmbed, a novel prediction model for N-linked glycosylation sites. We trained and tested StackGlyEmbed on two datasets, N-GlycositeAtlas and N-GlyDE. In determining a suitable representation of the residues, we have explored embeddings from several protein language models (ProtT5-XL-U50, ProteinBERT and ESM2) as well as physicochemical properties. We experimented with per-residue as well as window embeddings. As the datasets were imbalanced, we used an ensemble random undersampling approach for the base layer training with the training dataset. For the meta layer training, we used the validation set balanced using random undersampling. The base layer learners (SVM, XGB, and KNN) were chosen using incremental mutual information (IMI) analysis while SVM was chosen as meta learner based on the 10-Fold CV analysis on the validation set. The fact that the same optimal feature set and the same set of base learners were selected in both datasets is a testament to the robustness of our methodology and generalizability of the produced predictor. Indeed, StackGlyEmbed showed consistent and superior performance in both independent test sets, achieving 83.1% sensitivity, 69.3% F1-score, 51.5% MCC, and 65.9% AUPR on N-GlycositeAtlas-IT dataset, and 98.2% sensitivity, 89.1% F1-score, 82.6% MCC, and 93.1% AUPR on the N-GlyDE-IT dataset.

Although ensemble models are well-studied in bioinformatics, our work demonstrates the first application of the stacking framework in N-linked glycosylation prediction tasks confined to N-X-[S/T] sequons. The efficacy of stacking has been demonstrated through SHAP analysis, establishing BLP as the most important feature group. Another novelty of our work is the integration of both per-residue and per-window features derived from PLM embeddings, whereas most studies focusing on the N-X-[S/T] sequon have not. Windowing gives better localization as it considers the neighboring residues that may play a significant role in the glycosylation bindings. One of the final feature groups (ESM-2-Window) in our predictor utilizes windowing to have a positive impact on the prediction performance. Our model requires less expensive input data (embeddings from only protein sequences) than many state-of-the-art methods that require predicted or experimentally determined 3D protein structures as their input, despite the computational cost associated with using multiple PLMs. Because of this, our method works better with big datasets where structural information is more likely to be missing.

While researchers these days rush to build deep learning (DL) models for any prediction tasks, our results suggest that traditional ML models should not be ruled out. As a matter of fact, DL methods have been outperformed by traditional ML models in numerous recent bioinformatics studies (Liu *et al.* 2024, Rukh *et al.* 2024, Tran and Le 2024, Uddin *et al.* 2024, Yadav *et al.* 2024). Along that line, StackGlyEmbed too outperformed SOTA methods, many of which were based on deep learning architectures (LMNglyPred, DeepNGlyPred, NetNGlyc). Additionally, we also trained a feed-forward neural network (FNN) with our optimal set of feature groups on the N-GlycositeAtlas-TS and N-GlyDE-TS sets and respectively tested on N-GlycositeAtlas-IT and N-GlyDE-IT. The FNN delivered subpar performance compared to StackGlyEmbed, achieving 0.751 BACC, 0.669 F1, and 0.482 MCC on the N-GlycositeAtlas-IS set, and 0.894 BACC, 0.866 F1, and 0.785 MCC on the N-GlyDE-IS set. Deep learning models, including FNNs, typically require a significant amount of data to generalize well. The inferior performance of this FNN as well as SOTA DL methods could be due to the fact that DL models do not perform well under small datasets as they tend to have many parameters to learn, resulting in overfitting (Goodfellow *et al.* 2016).

Our work is not without limitations. The SHAP analysis had to be conducted on a reduced feature space to fit the computational requirements within our resources. A better, more informative analysis can be done on the actual feature space in future, given a considerable amount of computing resources. Another limitation is that our model does not consider the 3d structure of proteins. While it can produce improved prediction performance, it can also limit its applicability as novel proteins are sequenced at a much faster rate than their 3d structures are experimentally determined. To solve this quandary, predicted 3d structures from AlphaFold (Jumper *et al.* 2021) or alike could be used. However, the high computational demands and resource-intensive nature of such models prevented us from using predicted PDBs. For example, the generation of a predicted PDB file for a protein sequence with 1280 residues required ≈ 4 hours with 16 GB of memory, and 15 GB of VRAM on an Intel(R) Xeon(R) CPU @ 2.20 GHz and a Tesla T4 GPU. We recognize the potential of using structural features from predicted PDBs and leave that for future research. Given the results and standard practice, we maintained a single meta learner inside the stacking ensemble model architecture for this study. However, exploring multiple meta learners could be an avenue for future research.

StackGlyEmbed brings a substantial enhancement in the N-linked glycosylation site prediction task. It is freely available as open-source scripts at https://github.com/nafcoder/StackGlyEmbed. The prediction of glycosylation sites in novel proteins can be guided by this novel predictor. New glycosylation sites and their functional implications in biological systems may be discovered faster as a result. We genuinely hope that our easy-to-use and light-weight predictor will greatly aid in predicting N-linked glycosylation sites, thereby contributing to downstream tasks that depend on it, such as protein folding and stability, biological functions, immune responses, cell-to-cell communication, etc.

## Supplementary Material

vbaf146_Supplementary_Data

## Data Availability

Datasets, StackGlyEmbed model and scripts to reproduce the results are available at https://github.com/nafcoder/StackGlyEmbed.

## References

[vbaf146-B1] Hou X , Wang Y, Bu D et al EMNGly: predicting N-linked glycosylation sites using the language models for feature extraction. Bioinformatics 2023;39:btad650. 37930896 10.1093/bioinformatics/btad650 PMC10627407

[vbaf146-B2] Abdi H , Williams LJ. Principal component analysis. WIREs Comput Stats 2010;2:433–59.

[vbaf146-B3] Agarwal KL , Kenner GW, Sheppard RC. Feline gastrin. An example of peptide sequence analysis by mass spectrometry. J Am Chem Soc 1969;91:3096–7. 5784957 10.1021/ja01039a051

[vbaf146-B4] Altman DG , Bland JM. Diagnostic tests. 1: sensitivity and specificity. BMJ 1994;308:1552. 8019315 10.1136/bmj.308.6943.1552 PMC2540489

[vbaf146-B5] Brandes N , Ofer D, Peleg Y et al ProteinBERT: a universal deep-learning model of protein sequence and function. Bioinformatics 2022;38:2102–10. 35020807 10.1093/bioinformatics/btac020 PMC9386727

[vbaf146-B6] Chauhan JS , Bhat AH, Raghava GP et al GlycoPP: a webserver for prediction of N-and O-glycosites in prokaryotic protein sequences. PLoS One 2012;7:e40155. 22808107 10.1371/journal.pone.0040155 PMC3392279

[vbaf146-B7] Chauhan JS , Rao A, Raghava GP. In silico platform for prediction of N-, O-and C-glycosites in eukaryotic protein sequences. PLoS One 2013;8:e67008. 23840574 10.1371/journal.pone.0067008 PMC3695939

[vbaf146-B8] Chien C-H , Chang C-C, Lin S-H et al N-GlycoGo: predicting protein N-glycosylation sites on imbalanced data sets by using heterogeneous and comprehensive strategy. IEEE Access 2020;8:165944–50.

[vbaf146-B9] Chuang G-Y , Boyington JC, Joyce MG et al Computational prediction of N-linked glycosylation incorporating structural properties and patterns. Bioinformatics 2012;28:2249–55. 22782545 10.1093/bioinformatics/bts426 PMC3426846

[vbaf146-B10] Consortium U. UniProt: a worldwide hub of protein knowledge. Nucleic Acids Res 2019;47:D506–15. 30395287 10.1093/nar/gky1049 PMC6323992

[vbaf146-B11] Dang TH , Vu TA. xCAPT5: protein–protein interaction prediction using deep and wide multi-kernel pooling convolutional neural networks with protein language model. BMC Bioinform 2024;25:106. 10.1186/s12859-024-05725-6 PMC10924985 38461247

[vbaf146-B12] DeLano WL et al Pymol: an open-source molecular graphics tool. CCP4 Newsl Protein Crystallogr 2002;40:82–92.

[vbaf146-B13] Elnaggar A , Heinzinger M, Dallago C et al Prottrans: toward understanding the language of life through self-supervised learning. IEEE Trans Pattern Anal Mach Intell 2022;44:7112–27. 34232869 10.1109/TPAMI.2021.3095381

[vbaf146-B14] Fawcett T. An introduction to ROC analysis. Pattern Recognit Lett 2006;27:861–74.

[vbaf146-B15] Gavel Y , Heijne G. Sequence differences between glycosylated and non-glycosylated Asn-X-Thr/ser acceptor sites: implications for protein engineering. Protein Eng 1990;3:433–42. 2349213 10.1093/protein/3.5.433 PMC7529082

[vbaf146-B16] Goodfellow I , Bengio Y, Courville A. Deep Learning. Cambridge, MA: MIT Press, 2016. http://www.deeplearningbook.org.

[vbaf146-B17] Gupta R , Brunak S. Prediction of glycosylation across the human proteome and the correlation to protein function. In RB Altman, AK Dunker, L Hunter, K Lauerdale, TE Klein (Eds.), Biocomputing 2002. Singapore: World Scientific, 2001, 310–22. . 11928486

[vbaf146-B18] Gupta R , Jung E, Brunak S. Prediction of N-glycosylation sites in human proteins. Pac Symp Biocomput 2004;9:310–322. 11928486

[vbaf146-B19] Gurung BDS , Rayamajhi M, Baidya A et al Efficient prediction of Protein-Ligand binding using ESM-2 and Mol2vec with random Forest model. In: 2024 IEEE International Conference on Bioinformatics and Biomedicine (BIBM), pp. 6893–5. New York, NY: IEEE, 2024.

[vbaf146-B20] Huang Y , Niu B, Gao Y et al CD-HIT suite: a web server for clustering and comparing biological sequences. Bioinformatics 2010;26:680–2. 20053844 10.1093/bioinformatics/btq003 PMC2828112

[vbaf146-B21] Jumper J , Evans R, Pritzel A et al Highly accurate protein structure prediction with AlphaFold. Nature 2021;596:583–9. 34265844 10.1038/s41586-021-03819-2 PMC8371605

[vbaf146-B22] Keilwagen J , Grosse I, Grau J. Area under precision-recall curves for weighted and unweighted data. PLoS One 2014;9:e92209. 24651729 10.1371/journal.pone.0092209 PMC3961324

[vbaf146-B23] Kim Y , Kwon J. AttSec: protein secondary structure prediction by capturing local patterns from attention map. BMC Bioinform 2023;24:183. 10.1186/s12859-023-05310-3 PMC10161504 37142993

[vbaf146-B24] Kowarik M , Young NM, Numao S et al Definition of the bacterial N-glycosylation site consensus sequence. EMBO J 2006;25:1957–66. 16619027 10.1038/sj.emboj.7601087 PMC1456941

[vbaf146-B25] Li F , Li C, Wang M et al GlycoMine: a machine learning-based approach for predicting N-, C-and O-linked glycosylation in the human proteome. Bioinformatics 2015;31:1411–9. 25568279 10.1093/bioinformatics/btu852

[vbaf146-B26] Li W , Godzik A. Cd-hit: a fast program for clustering and comparing large sets of protein or nucleotide sequences. Bioinformatics 2006;22:1658–9. 16731699 10.1093/bioinformatics/btl158

[vbaf146-B27] Lin Z , Akin H, Rao R et al Evolutionary-scale prediction of atomic-level protein structure with a language model. Science 2023;379:1123–30. 36927031 10.1126/science.ade2574

[vbaf146-B28] Liu G , Chen X, Luan Y et al Viruspredictor: xgboost-based software to predict virus-related sequences in human data. Bioinformatics 2024;40:btae192. 38597887 10.1093/bioinformatics/btae192 PMC11052659

[vbaf146-B29] Lundberg SM , Lee S-I. A unified approach to interpreting model predictions. Adv Neural Inf Process Syst 2017:30:4765–74.

[vbaf146-B30] Marquet C , Heinzinger M, Olenyi T et al Embeddings from protein language models predict conservation and variant effects. Hum Genet 2022;141:1629–47. 34967936 10.1007/s00439-021-02411-y PMC8716573

[vbaf146-B31] Medzihradszky KF. Peptide sequence analysis. Methods Enzymol 2005;402:209–44. 16401511 10.1016/S0076-6879(05)02007-0

[vbaf146-B32] Mishra S , Misra O, Bhattacharya M. . Predicting the Protein-DNA binding residue using protein language model with residual based CNN module. In: 2024 IEEE International Conference on Bioinformatics and Biomedicine (BIBM), pp. 6127–6131. New York, NY: IEEE, 2024.

[vbaf146-B33] Nita-Lazar M , Wacker M, Schegg B et al The NXS/T consensus sequence is required but not sufficient for bacterial N-linked protein glycosylation. Glycobiology 2005;15:361–7. 15574802 10.1093/glycob/cwi019

[vbaf146-B34] Pakhrin SC , Aoki-Kinoshita KF, Caragea D et al DeepNGlyPred: a deep neural network-based approach for human N-linked glycosylation site prediction. Molecules 2021;26:7314. 34885895 10.3390/molecules26237314 PMC8658957

[vbaf146-B35] Pakhrin SC , Pokharel S, Aoki-Kinoshita KF et al LMNglyPred: prediction of human N-linked glycosylation sites using embeddings from a pre-trained protein language model. Glycobiology 2023;33:411–22. 37067908 10.1093/glycob/cwad033

[vbaf146-B36] Palmisano G , Melo-Braga MN, Engholm-Keller K et al Chemical deamidation: a common pitfall in large-scale N-linked glycoproteomic mass spectrometry-based analyses. J Proteome Res 2012;11:1949–57. 22256963 10.1021/pr2011268

[vbaf146-B37] Petrescu A-J , Milac A-L, Petrescu SM et al Statistical analysis of the protein environment of N-glycosylation sites: implications for occupancy, structure, and folding. Glycobiology 2004;14:103–14. 14514716 10.1093/glycob/cwh008

[vbaf146-B38] Pitti T , Chen C-T, Lin H-N et al N-GlyDE: a two-stage N-linked glycosylation site prediction incorporating gapped dipeptides and pattern-based encoding. Sci Rep 2019;9:15975. 31685900 10.1038/s41598-019-52341-z PMC6828726

[vbaf146-B39] Powers DM. Evaluation: from precision, recall and F-measure to ROC, informedness, markedness and correlation. arXiv 2020. https://arxiv.org/abs/2010.16061, preprint: not peer reviewed.

[vbaf146-B40] Pugalenthi G , Nithya V, Chou K-C et al Nglyc: a random Forest method for prediction of N-glycosylation sites in eukaryotic protein sequence. Protein Pept Lett 2020;27:178–86. 31577193 10.2174/0929866526666191002111404

[vbaf146-B41] Ramazi S , Zahiri J. Post-translational modifications in proteins: resources, tools and prediction methods. Database, 2021;2021:baab012. 33826699 10.1093/database/baab012 PMC8040245

[vbaf146-B42] Rukh G , Akbar S, Rehman G et al StackedEnC-AOP: prediction of antioxidant proteins using transform evolutionary and sequential features based multi-scale vector with stacked ensemble learning. BMC Bioinform 2024;25:256. 10.1186/s12859-024-05884-6 PMC11298090 39098908

[vbaf146-B43] Schmirler R , Heinzinger M, Rost B. Fine-tuning protein language models boosts predictions across diverse tasks. Nat Commun 2024;15:7407. 39198457 10.1038/s41467-024-51844-2 PMC11358375

[vbaf146-B44] Schulz BL. Beyond the sequon: sites of N-glycosylation. In: S Petrescu (ed.). Glycosylation. Rijeka: InTech, 2012, 21–40.

[vbaf146-B45] Simon E , Bankapur SS. Prediction of drug-target interactions using BERT for protein sequences and drug compound. In: 2024 16th International Conference on COMmunication Systems & NETworkS (COMSNETS). 2024, pp. 436–8. Piscataway, NJ: IEEE.

[vbaf146-B46] Slade DJ , Subramanian V, Fuhrmann J et al Chemical and biological methods to detect post-translational modifications of arginine. Biopolymers 2014;101:133–43. 23576281 10.1002/bip.22256 PMC3900596

[vbaf146-B47] Sun S , Hu Y, Ao M et al N-GlycositeAtlas: a database resource for mass spectrometry-based human N-linked glycoprotein and glycosylation site mapping. Clin Proteomics 2019;16:35–11. 31516400 10.1186/s12014-019-9254-0 PMC6731604

[vbaf146-B48] Sun X , Wu Z, Su J et al GraphPBSP: protein binding site prediction based on graph attention network and pre-trained model ProstT5. Int J Biol Macromol 2024;282:136933. 39471921 10.1016/j.ijbiomac.2024.136933

[vbaf146-B49] Susanty M , Mursalim MKN, Hertadi R et al Classifying alkaliphilic proteins using embeddings from protein language model. Comput Biol Med 2024;173:108385. 38547659 10.1016/j.compbiomed.2024.108385

[vbaf146-B50] Taherzadeh G , Dehzangi A, Golchin M et al SPRINT-Gly: predicting N-and O-linked glycosylation sites of human and mouse proteins by using sequence and predicted structural properties. Bioinformatics 2019;35:4140–6. 30903686 10.1093/bioinformatics/btz215

[vbaf146-B51] Thumuluri V , Almagro AJJ, Johansen AR et al DeepLoc 2.0: multi-label subcellular localization prediction using protein language models. Nucleic Acids Res 2022;50:W228–W234. 35489069 10.1093/nar/gkac278 PMC9252801

[vbaf146-B52] Tran T-O , Le NQK. Sa-ttca: an SVM-based approach for tumor t-cell antigen classification using features extracted from biological sequencing and natural language processing. Comput Biol Med 2024;174:108408. 38636332 10.1016/j.compbiomed.2024.108408

[vbaf146-B53] Uddin I , Awan HH, Khalid M et al A hybrid residue based sequential encoding mechanism with XGBoost improved ensemble model for identifying 5-hydroxymethylcytosine modifications. Sci Rep 2024;14:20819. 39242695 10.1038/s41598-024-71568-z PMC11379919

[vbaf146-B54] Vergara JR , Estévez PA. A review of feature selection methods based on mutual information. Neural Comput Applic 2014;24:175–86.

[vbaf146-B55] Wacker M , Feldman MF, Callewaert N et al Substrate specificity of bacterial oligosaccharyltransferase suggests a common transfer mechanism for the bacterial and eukaryotic systems. Proc Nat Acad Sci USA 2006;103:7088–93. 16641107 10.1073/pnas.0509207103 PMC1459022

[vbaf146-B56] Wang H , Zhao L, Yu Z et al CoNglyPred: accurate prediction of N-Linked glycosylation sites using ESM-2 and structural features with graph network and Co-Attention. Proteomics 2025;25:e202400210. 39361250 10.1002/pmic.202400210

[vbaf146-B57] Wu S , Xu J, Guo J-T. Prediction of single-stranded DNA binding proteins with protein language model. In: 2024 IEEE International Conference on Bioinformatics and Biomedicine (BIBM). 2024, pp. 258–63. New York, NY: IEEE.

[vbaf146-B58] Xia C-Q , Pan X, Shen H-B. Protein–ligand binding residue prediction enhancement through hybrid deep heterogeneous learning of sequence and structure data. Bioinformatics 2020;36:3018–27. 32091580 10.1093/bioinformatics/btaa110

[vbaf146-B59] Yadav AK , Gupta PK, Singh TR. PMTPred: machine-learning-based prediction of protein methyltransferases using the composition of k-spaced amino acid pairs. Mol Divers 2024;28:2301–15. 39033257 10.1007/s11030-024-10937-2

[vbaf146-B60] Yeo I-K , Johnson RA. A new family of power transformations to improve normality or symmetry. Biometrika 2000;87:954–9.

[vbaf146-B61] Zielinska DF , Gnad F, Wiśniewski JR et al Precision mapping of an in vivo N-glycoproteome reveals rigid topological and sequence constraints. Cell 2010;141:897–907. 20510933 10.1016/j.cell.2010.04.012

