# Supplementary material for: StackGlyEmbed: prediction of N-linked glycosylation sites using protein language models
Source: Bioinform Adv. 2025 Jun 28;5(1):vbaf146. doi: 10.1093/bioadv/vbaf146 (PMC12237515; doi:10.1093/bioadv/vbaf146)
Supplement: vbaf146_Supplementary_Data [file vbaf146_supplementary_data.zip › OUP_StackGlyEmbed_Supplementary.pdf]

Supplementary information: StackGlyEmbed: Prediction of N-linked Glycosylation sites using protein language models

Md Muhaiminul Islam Nafi[1,2] and M Saifur Rahman[1,3]

[1]Department of CSE, BUET, Dhaka 1000, Bangladesh
[2]Department of CSE, United International University (UIU), Dhaka 1212, Bangladesh
[3]Corresponding author: Tel: +8801715010010, Email: mrahman@cse.buet.ac.bd

June 16, 2025

Supplementary Material

This supplementary section provides additional tables.

Table S1: 10-Fold CV performance of all three ProteinBERT derived feature groups, averaged over all 10 traditional ML models, trained on the N-GlycositeAtlas-TS set that was balanced using random undersampling.

**Feature group**	**SN**	**SP**	**BACC**	**ACC**	**PREC**	**F1**	**MCC**	**AUC**	**AUROC**
ProteinBERT-Per-Residue	0.658	0.608	0.633	0.633	0.626	0.641	0.266	0.68	0.646
ProteinBERT-Window	0.711	0.63	0.67	0.67	0.659	0.683	0.343	0.726	0.691
ProteinBERT	**0.794**	**0.774**	**0.784**	**0.784**	**0.781**	**0.787**	**0.568**	**0.85**	**0.826**

Table S2: 10-Fold CV performance of all three ProteinBERT derived feature groups, averaged over all 10 traditional ML models, trained on the N-GlyDE-TS set that was balanced using random undersampling.

**Feature group**	**SN**	**SP**	**BACC**	**ACC**	**PREC**	**F1**	**MCC**	**AUC**	**AUROC**
ProteinBERT-Per-Residue	0.668	0.626	0.647	0.647	0.641	0.653	0.296	0.701	0.676
ProteinBERT-Window	**0.764**	0.673	0.718	0.718	0.703	**0.73**	0.441	**0.777**	0.738
ProteinBERT	0.689	**0.755**	**0.722**	**0.722**	**0.74**	0.712	**0.447**	0.762	**0.747**

Table S3: 10-Fold CV results for hyperparameter tuning of the SVM meta-classifier on the N-GlycositeAtlas-TS dataset across different window sizes: 21, 27, 31, 37, and 41.

**Window**	**SN**	**SP**	**BACC**	**ACC**	**PREC**	**F1**	**MCC**	**AUC**	**AUROC**
Window_21	0.861	0.747	0.804	0.804	0.773	0.815	0.612	0.876	0.851
Window_27	0.863	**0.75**	**0.807**	**0.807**	**0.775**	**0.817**	0.617	0.879	0.853
Window_31	0.86	**0.75**	0.805	0.805	**0.775**	0.815	0.614	**0.88**	**0.855**
Window_37	0.857	0.746	0.802	0.802	0.771	0.812	0.607	0.878	0.853
Window_41	**0.864**	**0.75**	**0.807**	**0.807**	**0.775**	**0.817**	**0.618**	0.879	0.854

Table S4: 10-Fold CV results for hyperparameter tuning of the SVM meta-classifier on the N-GlyDE-TS dataset across different window sizes: 21, 27, 31, 37, and 41.

**Window**	**SN**	**SP**	**BACC**	**ACC**	**PREC**	**F1**	**MCC**	**AUC**	**AUROC**
Window_21	0.949	0.755	0.852	0.852	0.795	0.865	0.718	0.912	0.89
Window_27	0.949	0.757	0.853	0.853	0.796	0.866	0.72	0.913	0.893
Window_31	**0.951**	**0.76**	**0.856**	**0.856**	**0.799**	**0.868**	**0.725**	**0.918**	**0.901**
Window_37	0.947	0.753	0.85	0.85	0.793	0.863	0.713	0.916	0.897
Window_41	0.944	0.757	0.851	0.851	0.796	0.864	0.714	0.917	0.9

Table S5: Performance of models trained on the N-GlycositeAtlas-TS set and tested on the N-GlyDE-IT set

**Models**	**SN**	**SP**	**BACC**	**ACC**	**PREC**	**F1**	**MCC**	**AUROC**	**AUC**
StackGlyEmbed	0.47	**0.582**	0.526	**0.54**	**0.4**	0.432	0.051	**0.539**	0.363
LMNglyPred	**0.681**	0.374	**0.527**	0.489	0.394	**0.499**	**0.055**	0.527	**0.387**

Table S6: Performance of models trained on the N-GlyDE-TS set and tested on the N-GlycositeAtlas-IT set

**Models**	**SN**	**SP**	**BACC**	**ACC**	**PREC**	**F1**	**MCC**	**AUROC**	**AUC**
StackGlyEmbed	**0.618**	0.489	**0.554**	0.532	**0.379**	**0.47**	**0.102**	**0.583**	**0.396**
LMNglyPred	0.467	**0.615**	0.541	**0.565**	**0.379**	0.419	0.079	0.541	0.356

Table S7: Performance of StackGlyEmbed trained on the merged non-redundant training set and evaluated on the merged non-redundant test set. The training and test sets were created by merging the N-GlyDE-TS and N-GlycositeAtlas-TS training sets, and the N-GlyDE-IT and N-GlycositeAtlas-IT test sets, respectively. Sequence redundancy was reduced using CD-HIT at a 30% sequence similarity threshold.

**Predictor**	**SN**	**SP**	**BACC**	**ACC**	**PREC**	**F1**	**MCC**	**AUC**	**AUROC**
StackGlyEmbed	0.842	0.681	0.762	0.733	0.554	0.669	0.488	0.809	0.541
